# Exploring the Clinical Value of Perioperative ctDNA‐Based Detection of Molecular Residual Disease in Patients With Esophageal Squamous Cell Carcinoma

**DOI:** 10.1111/1759-7714.70017

**Published:** 2025-02-18

**Authors:** Jimin Li, Congcong Wu, Yongming Song, Yuhui Fan, Chao Li, Haibo Li, Shuangping Zhang

**Affiliations:** ^1^ Department of Thoracic Surgery Cancer Hospital Affiliated to Shanxi Medical University, Shanxi Province Cancer Hospital, Shanxi Hospital Affiliated to Cancer Hospital, Chinese Academy of Medical Sciences Taiyuan China; ^2^ Department of Thoracic Surgery Shanxi Province Cancer Hospital, Shanxi Hospital Affiliated to Cancer Hospital, Chinese Academy of Medical Sciences, Cancer Hospital Affiliated to Shanxi Medical University Taiyuan China; ^3^ Department of Thoracic Surgery Jincheng Second People's Hospital Jincheng China; ^4^ Department of Thoracic Surgery Yuncheng Central Hospital Yuncheng China

**Keywords:** ctDNA, ESCC, molecular residual disease

## Abstract

**Objective:**

To explore the clinical value of molecular residual disease detection based on circulating tumor DNA (ctDNA‐MRD) in the perioperative period of esophageal squamous cell carcinoma (ESCC) and to analyze the tumor escape mechanisms in MRD‐positive cases.

**Methods:**

A total of 35 ESCC patients were prospectively enrolled. Preoperative and postoperative (1 month after surgery) blood and surgical tissue samples were analyzed. ctDNA variants were tracked in plasma to assess ctDNA‐MRD, and whole‐transcriptome sequencing was performed on MRD‐positive and MRD‐negative tissue samples.

**Results:**

Preoperative blood ctDNA was positive in 54.3% of patients, with a 31.6% positive predictive value for recurrence. One month postsurgery, the positive rate of ctDNA was 17.1%, with an 83.3% predictive value for recurrence. Both preoperative and postoperative ctDNA positivity were significant prognostic indicators (HR = 2.78, *p* < 0.05; HR = 4.42, *p* < 0.001). Multivariate analysis confirmed ctDNA as an independent prognostic factor (HR = 303.75, *p* < 0.001). Transcriptomic analysis revealed increased macrophage (W = 15 848; *p* < 0.01) and follicular helper T (Tfh) cell (W = 10 935; *p* < 0.01) levels in MRD‐positive patients, suggesting a potential link to immune escape in tumors.

**Conclusions:**

Plasma ctDNA measured 1 month postoperatively in ESCC patients can effectively detect MRD, and ctDNA‐MRD serves as an independent risk factor for postoperative recurrence. The mechanism underlying MRD positivity may involve the polarization of Tfh cells and macrophages, aiding tumor cells in immune escape through the bloodstream.

## Graphical abstract

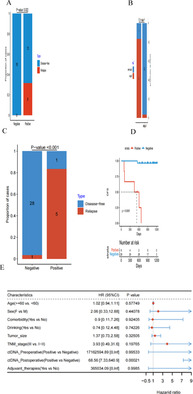

## 1 Introduction

Esophageal cancer is one of the most common malignant tumors of the digestive tract worldwide, characterized by high malignancy, incidence, and mortality rates, leading to poor prognosis [1]. China is a high‐incidence area for esophageal cancer, with esophageal squamous cell carcinoma (ESCC) predominating, accounting for over 90% of cases. Currently, the treatment of esophageal cancer has evolved into a comprehensive approach [2, 3] centered on surgical intervention, supplemented by radiotherapy, chemotherapy, and immunotherapy. As shown in the CheckMate 577 study [4], disease‐free survival (DFS) was significantly longer among patients with resected esophageal or gastroesophageal junction cancer who received neoadjuvant chemoradiotherapy who received adjuvant nivolumab than among those who received placebo Despite the improvement of diagnostic techniques, surgical skills and prognostic indicators for esophageal cancer [5, 6], the treatment of esophageal cancer remains unsatisfactory. The overall 5‐year survival rate for patients with ESCC in China is still below 30% [7], posing a serious threat to the health of the Chinese population.

In recent years, the emergence of molecular residual disease detection based on circulating tumor DNA (ctDNA‐MRD) detection technology has provided new avenues for cancer clinical treatment [8, 9]. By accurately identifying the residual tumor status in patients, ctDNA‐MRD detection not only aids in targeted clinical interventions for high‐risk cancer patients, enhancing treatment efficacy, but also reduces unnecessary treatments for low‐risk patients, thereby lessening physical and financial burdens. This technology has achieved significant research outcomes across various cancer domains. In the field of lung cancer, research by Zhang et al. [10] demonstrated a strong correlation between MRD and clinical outcomes through ctDNA‐MRD detection. Their findings suggest that patients potentially curable (with undetectable MRD upon longitudinal testing) and those who may not require adjuvant therapy (with undetectable MRD) can be identified. In colorectal cancer, a study by Kotani et al. [11] indicated that postoperative ctDNA status serves as a more critical prognostic biomarker compared to currently used high‐risk clinicopathological features and may predict the benefits of adjuvant chemotherapy (ACT). In the realm of lymphoma, research by Fernández‐Miranda et al. [12] revealed that baseline ctDNA levels are associated with the risk of early progression and treatment response in follicular lymphoma. Monitoring circulating free DNA (cfDNA) and genotyping during treatment and follow‐up can predict therapeutic response and early progression.

To date, research on ctDNA in ESCC remains limited. This study prospectively enrolled ESCC patients, collecting their tumor tissues and matched plasma samples. Using next‐generation sequencing (NGS), we performed whole‐transcriptome sequencing on both MRD‐positive and MRD‐negative patients. The objective is to predict postoperative recurrence risk by analyzing perioperative ctDNA‐MRD results and to explore the underlying mechanisms.

## 2 Materials and Methods

### 2.1 Clinical Data

We prospectively collected clinical data from 35 patients with ESCC who underwent radical surgery in the Department of Thoracic Surgery at Shanxi Cancer Hospital between January 2021 and January 2023. Samples collected included surgical tissue, preoperative peripheral blood, and peripheral blood at 1 month postoperatively.

#### 2.1.1 Inclusion Criteria

Pathologically confirmed ESCC with radical surgery performed. Complete clinical information available, with collected samples of preoperative peripheral blood, surgical tissue, and peripheral blood at 1 month ± 1 week postoperatively.

#### 2.1.2 Exclusion Criteria

History of other tumors within the past 5 years. Missing clinical, pathological, or follow‐up data. This study complies with the relevant requirements of the Declaration of Helsinki and has been approved by the Ethics Committee of Shanxi Cancer Hospital (Approval No: 2022JC07). All patients signed informed consent forms.

### 2.2 Experimental Methods

#### 2.2.1 DNA Panel Sequencing

Peripheral blood samples were centrifuged at 1600 × *g* for 15 min at 4°C. The supernatant was transferred to a centrifuge tube and further centrifuged at 12 000 × *g* for 15 min. Plasma and leukocytes were separately stored at −80°C. DNA was extracted from leukocytes and tissue samples, and fragmented to approximately 200 bp using a Covaris ultrasonic disruptor (Covaris, USA). Libraries were constructed using the SureSelect XT HS Kit (Agilent Technologies, USA) or KAPA HyperPrep Kit (Roche, Switzerland) with 5 ng cfDNA and 200 ng gDNA. Both gDNA and cfDNA were captured using a panel targeting 1123 genes and sequenced on the MGISEQ‐2000 platform (MGI Tech, Shenzhen, China). Sequencing data volumes were 5 Gb for leukocytes, 10 Gb for tissue samples, and 40 Gb for plasma cfDNA.

#### 2.2.2 Transcriptome Sequencing

RNA was extracted from tissue samples using the RNeasy FFPE Kit (QIAGEN, Germany) and quantified with the Qubit RNA HS Assay Kit (Thermo Fisher Scientific, USA). For library preparation, 50 ng of RNA was reverse transcribed into cDNA, followed by adapter ligation and rRNA depletion. All libraries were enriched through 13 cycles of amplification on a SimpliAmp Thermal Cycler (Thermo Fisher Scientific, USA) and subsequently sequenced on the Illumina NovaSeq 6000 platform (Illumina, USA).

#### 2.2.3 Tumor Tissue Mutation Analysis

Somatic mutations in tumor tissues were analyzed using the integrated software GATK and VarScan. Detailed mutation filtering parameters were referenced from previously reported literature [11].

#### 2.2.4 MRD Analysis

Sequencing was performed on available tumor tissue samples to identify specific tumor mutations. Plasma mutations were tracked based on variations detected in the corresponding tissue samples, employing two different methods to assess the presence of tissue‐matched mutations in plasma. Mutations originating from tissue in plasma were considered reliable if they showed statistically significant differences from background error. Tumor‐specific driver mutations required support from at least two reads with a mapping quality > 30, while other nondriver gene mutations required support from at least five reads with a mapping quality > 30. If at least one tissue‐tracked site is detected as positive in plasma, it is defined as ctDNA‐positive or MRD‐positive. Conversely, it is defined as ctDNA‐negative or MRD‐negative.

#### 2.2.5 Transcriptome Analysis

Sequencing adapters and low‐quality bases were removed using Trimmomatic (version 0.32). Subsequently, sequences were aligned to the human reference genome (hg19) using the Subread package with default parameters. Gene expression quantification was performed by aggregating read counts for UCSC RefSeq genes using the FeatureCounts program from Subread. Normalization of read counts and differential gene expression (DEG) analysis were conducted using DESeq2. DEGs were identified based on the criteria of *p* < 0.05 and |log2(fold change)| > 1. Pathway enrichment analysis of candidate DEGs was performed using the enrichKEGG function from the R package ClusterProfiler, with a significance threshold of *p* < 0.01.

#### 2.2.6 Statistical Analysis

Statistical analyses and data visualization were conducted using R/Bioconductor packages. Comparisons of immune infiltration between two groups were performed using the Wilcoxon rank‐sum test. Associations between clinical recurrence and preoperative or postoperative ctDNA status were evaluated using Fisher's exact test. Overall survival (OS) curves were generated using the Kaplan–Meier method, with group comparisons made via the log‐rank test. A *p* value < 0.05 was considered statistically significant. Multivariate Cox regression analysis was employed to assess the impact of multiple factors on survival time. All analyses were conducted in R version 4.1.3 (https://www.r‐project.org/) using the survival, survminer, and ggsignif packages.

## 3 Results

### 3.1 Baseline Molecular Characteristics of ESCC Patients

This multicenter study enrolled 35 operable ESCC patients with a follow‐up period of 2 years; their clinical information is presented in Table 1. All enrolled patients underwent NGS, revealing the following molecular characteristics.

**TABLE 1:** Statistical table of clinical information of enrolled subjects.

Characteristic	Number (%)
Median age (range)	57.0 (43–77)
Gender	
Female	17 (48.5%)
Male	18 (51.5%)
Alcohol consumption	
Yes	14 (40%)
No	21 (60%)
Tumor size (median, range)	3 cm (1.3–7 cm)
TNM stage	
I	6 (17.1%)
II	19 (54.3%)
III	10 (28.6%)
Adjuvant therapy	
Yes	29 (82.9%)
No	6 (17.1%)
Preoperative ctDNA status	
Positive	19 (54.3%)
Negative	16 (45.7%)
Postoperative ctDNA‐MRD status	
Positive	29 (82.9%)
Negative	6 (17.1%)

#### 3.1.1 Gene Mutations

**FIGURE 1:**
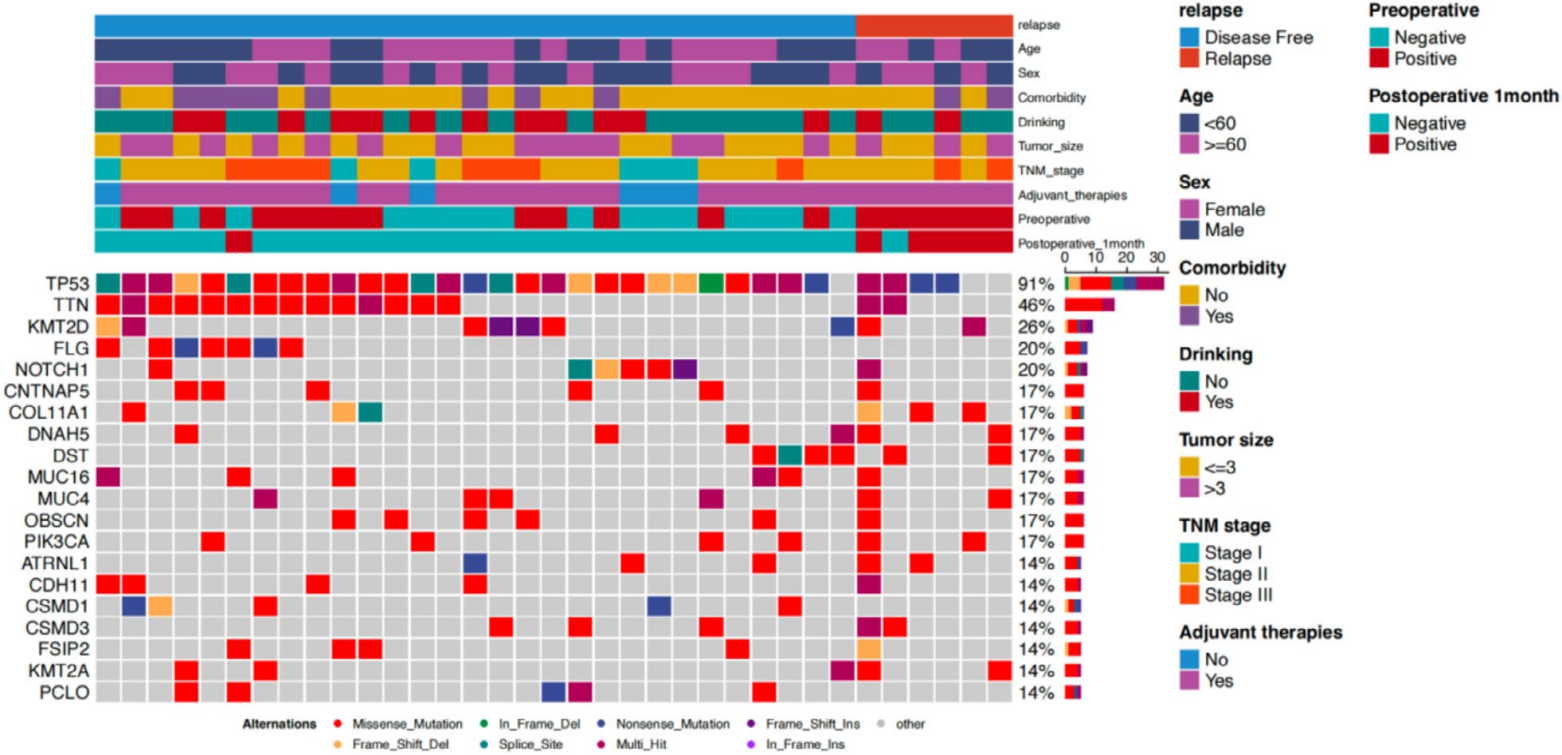
Baseline variation map of 35 enrolled patients.

Frequent mutations were observed in genes such as TP53, KMT2D, and NOTCH1 (see Figure 1).

#### 3.1.2 Structural Variations

ESCC exhibited specific patterns of gene amplifications and deletions. Common amplification regions included those encompassing genes like CCND1 and MYC.

#### 3.1.3 Signaling Pathways

The development and progression of ESCC are closely associated with aberrant activities in multiple signaling pathways, including cell cycle and apoptosis regulation, PI3K/mTOR, Notch, and Wnt/β‐catenin pathways.

### 3.2 Preoperative ctDNA Predicts Recurrence Risk in ESCC Patients

**FIGURE 2:**
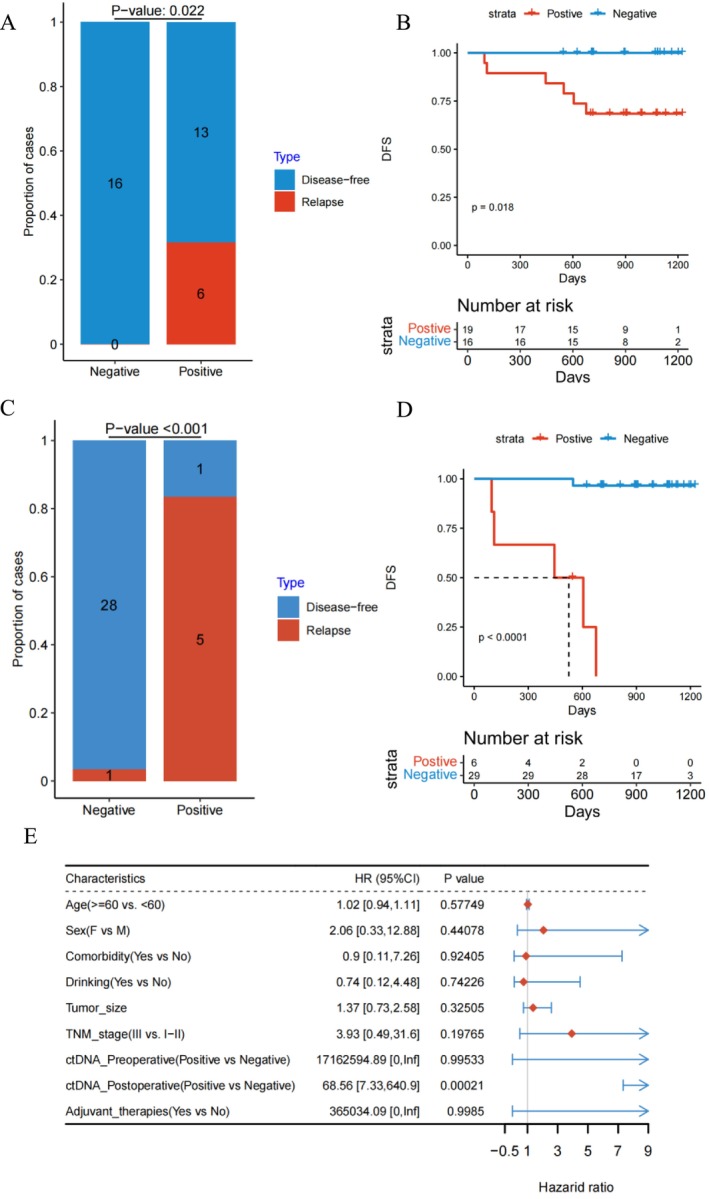
Preoperative ctDNA value of ESCC: Relationship between clinical recurrence and preoperative blood (A) and postoperative blood (C) ctDNA status; The predictive results of preoperative blood (B) and postoperative blood (D) ctDNA status for prognosis; Multivariate analysis of prognostic indicators (E).

Analysis of preoperative plasma ctDNA tracking tissue mutations revealed a ctDNA positivity rate of 54.3% (19 out of 35). The sensitivity, specificity, and positive predictive value of ctDNA status in predicting recurrence were 100%, 55.2%, and 31.6%, respectively. There was a significant difference in recurrence between ctDNA‐negative and ctDNA‐positive patients (*p* = 0.022; see Figure 2A). To assess the relationship between preoperative ctDNA status and prognosis, we conducted the Kaplan–Meier survival analysis. The results indicated that preoperative plasma ctDNA status effectively distinguished prognosis (HR = 2.78, 95% CI [2.05–20.55]; *p* < 0.05), with ctDNA‐positive patients exhibiting poorer outcomes (see Figure 2B).

### 3.3 Postoperative ctDNA MRD as an Independent Prognostic Factor

We evaluated the status of ctDNA MRD 1 month postoperatively. The analysis revealed a recurrence rate of 83.3% (five out of six) in patients with positive ctDNA MRD, with sensitivity and specificity of 83.3% and 96.6%, respectively. There was a significant difference in recurrence between ctDNA‐negative and ctDNA‐positive patients (OR = 88.91, *p* < 0.001; see Figure 2C). Patients with positive MRD had significantly poorer prognosis compared to negative patients (HR = 4.42, 95% CI [3.5–33.86]; *p* < 0.001; see Figure 2D). Multivariate analysis identified positive MRD as an independent high‐risk factor for postoperative recurrence in ESCC (*p* < 0.001; see Figure 2E).

### 3.4 Differential Expression Between MRD‐Positive and MRD‐Negative Patients

**FIGURE 3:**
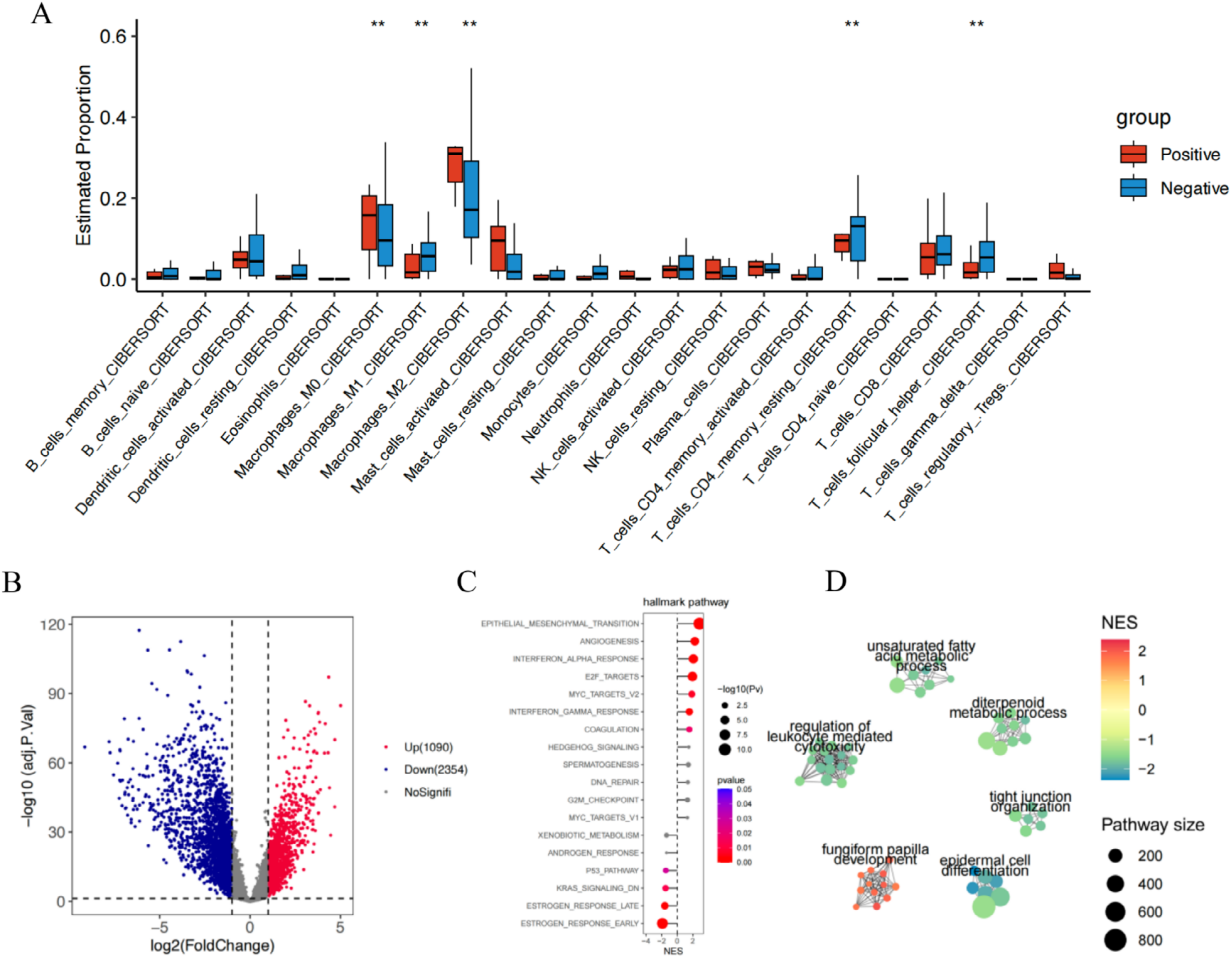
The figure illustrates the differential expression between MRD‐negative and MRD‐positive patients. (A) Differences in the tumor microenvironment between MRD‐negative and MRD‐positive patients. (B) DEGs between the two groups. (C) Hallmark pathway enrichment results of the DEGs. (D) Clustering results of the enriched pathways.

We performed transcriptome sequencing on MRD‐positive patients and matched MRD‐negative patients. The analysis revealed significant differences in the tumor microenvironment between MRD‐positive and MRD‐negative patients, particularly in macrophages (*W* = 15 848; *p* < 0.01), resting CD4 memory T cells (*W* = 13 249; *p* < 0.01), and Tfh cells (*W* = 10 935; *p* < 0.01) (Figure 3A). A total of 3444 differentially expressed genes (DEGs) were identified between the two groups, with 1090 genes showing upregulation (Figure 3B). These DEGs were primarily enriched in pathways related to epithelial–mesenchymal transition and angiogenesis (Figure 3C). Further gene ontology clustering analysis indicated that these pathways were mainly associated with processes such as unsaturated fatty acid metabolism and epidermal cell differentiation (Figure 3D).

## 4 Discussion

Esophageal cancer is one of the most prevalent and lethal malignancies worldwide [13]. In China, ESCC constitutes the predominant pathological type, accounting for over 90% of all esophageal cancer cases [14, 15]. Currently, the postoperative recurrence risk of esophageal cancer remains high. Traditional imaging modalities and tumor markers, such as carcinoembryonic antigen (CEA), squamous cell carcinoma (SCC) antigen, Cytokeratin 19 Fragment Antigen 21‐1 (CYFRA21‐1), exhibit limited sensitivity and are often delayed in predicting recurrence. Therefore, exploring new and more effective biomarkers is essential for the personalized and precise management of esophageal cancer [16, 17]. Studies have demonstrated that detecting ctDNA‐MRD in plasma holds unique clinical value [18, 19], enabling the early prediction of postoperative recurrence risk [16]. However, research on the perioperative use of ctDNA‐MRD to predict recurrence risk in ESCC is limited, highlighting an urgent clinical need to understand its value in ESCC patients. To date, ctDNA‐MRD research in lung cancer has matured, providing valuable insights for clinical decision‐making [20]. In the field of esophageal cancer, the current treatment paradigm centers on surgery, complemented by perioperative comprehensive management and recurrence monitoring for early and mid‐stage disease. Research on ctDNA as a monitoring indicator is still in its infancy.

Azad et al. [16] demonstrated that ctDNA is associated with tumor progression, distant metastasis, and shorter survival times. Compared to imaging, ctDNA detection can identify distant metastases earlier. In patients receiving chemoradiotherapy without surgery, combined ctDNA and metabolic imaging analysis predicted progression in 100% of patients with tumor progression. A prospective study conducted by Ng et al. [21] found that high ctDNA levels at 6 months postoperatively in ESCC patients undergoing radical surgery were associated with poor OS. In patients receiving neoadjuvant chemoradiotherapy, changes in ctDNA after treatment were associated with poor progression‐free survival (PFS). In the palliative treatment group, changes in nuclear factor erythroid 2‐related factor 2 (NFE2L2) before chemotherapy were independently associated with PFS, whereas high ctDNA levels and ctDNA changes before the third cycle showed weaker associations with PFS. However, ctDNA changes before chemotherapy were independently associated with OS. The proposed prognostic model incorporating ctDNA features is useful in ESCC, and ctDNA levels and NFE2L2 changes are important prognostic factors. Changes in ctDNA after radical treatment may indicate a high risk of recurrence, and high‐risk patients may benefit from timely treatment modifications.

This study is a prospective investigation into perioperative monitoring of ctDNA‐MRD in ESCC, showing that perioperative ctDNA‐MRD monitoring helps assess recurrence risk and provides critical guidance on the need for adjuvant therapy. The results indicate that both preoperative blood ctDNA and postoperative blood ctDNA‐MRD effectively distinguish the recurrence risk following radical surgery for ESCC, with postoperative ctDNA‐MRD identified as an independent prognostic factor for recurrence. Liu et al. [22] found that ctDNA positivity in plasma 1‐week postsurgery has a more significant impact on DFS and OS than any other clinicopathological risk factors. Patients with postoperative ctDNA positivity exhibited notably shorter DFS, serving as an independent prognostic factor. Related studies [23] suggest that trauma induces an initial cfDNA peak after surgery, with levels taking about 4 weeks to return to preoperative baselines. High cfDNA levels may hinder ctDNA detection, potentially masking ctDNA positivity in patients with recurrence. Therefore, measuring ctDNA in plasma 1‐month postsurgery offers a more accurate reflection of ctDNA status, further affirming postoperative ctDNA‐MRD as an independent risk factor for recurrence. The research by Ococks et al. [24] demonstrated that plasma ctDNA post‐esophagectomy in esophageal adenocarcinoma predicts recurrence. The present study confirms that ctDNA effectively predicts postoperative recurrence risk in ESCC patients, providing additional evidence for the role of ctDNA in esophageal cancer monitoring and further validating its clinical value in this field.

The study by Verschoor et al. [25] confirmed that in multiple cancer types, including esophageal cancer, non‐small cell lung cancer, colorectal cancer (CRC), and breast cancer, patients who received adjuvant therapy guided by blood ctDNA had a better prognosis than those who did not, and longitudinal ctDNA monitoring (particularly in tumors with lower proliferative activity) exhibited high sensitivity in predicting postoperative recurrence. The prospective multicenter study by Reinert et al. [26] found that CRC patients who were ctDNA‐positive postoperatively had a significantly higher risk of recurrence compared to ctDNA‐negative patients, and this risk further increased after ACT 0.30% of ctDNA‐positive patients achieved clearance through ACT. ctDNA analysis holds promise for risk stratification in postoperative CRC management, monitoring of ACT, and early detection of recurrence. Therefore, based on the current findings of this study, it can be speculated that adjuvant therapy for postoperative ctDNA‐positive patients may improve their prognosis, and further research will be conducted in the future. Nowadays, detecting MRD using ctDNA to guide clinical treatment strategies has become one of the hottest topics in oncology research [27]. ctDNA‐based MRD detection enables early identification of recurrent patients, the development of personalized treatment plans, and prognosis assessment. However, there are still knowledge gaps regarding the optimal detection methods, the definition of MRD (including MRD thresholds and detection techniques, which remain unstandardized), and personalized treatment strategies. In the future, the development of more sensitive detection technologies, the establishment of standardized MRD definitions, and in‐depth research into personalized treatment strategies will help refine clinical guidelines and ultimately extend patient survival.

The mechanisms of tumor immune evasion are complex, with studies indicating that the tumor microenvironment plays a significant role in this process [28, 29]. Analysis of the tumor microenvironment in MRD‐positive patients reveals elevated expression of hallmark pathways such as epithelial–mesenchymal transition and angiogenesis, which can promote tumor metastasis and recurrence, serving as indicators of disease progression [30]. Our study shows that MRD‐positive patients exhibit higher expression of macrophages and Tfh cells. This may be related to the increased involvement of Tfh cells in humoral immune responses and their role in promoting tumor immune evasion through macrophages.

This study is a prospective study with a relatively small overall sample size, and the MRD results may have some variability. Additionally, the regulatory mechanism of MRD+ immune escape has not been further explored in depth. Further studies with larger sample sizes are needed to investigate the specific mechanisms of esophageal cancer recurrence, metastasis, and tumor immune escape. The role of ctDNA as a longitudinal marker to guide adjuvant therapy has not yet been validated. In the future, its significance as a reference for adjuvant treatment planning can be verified by incorporating retrospective ctDNA data from randomized controlled trials.

In summary, plasma ctDNA measured 1 month postoperatively in ESCC patients can effectively detect MRD, and ctDNA‐MRD serves as an independent risk factor for postoperative recurrence. MRD‐positive patients exhibit elevated expression of macrophages and Tfh cells, which may be crucial factors in tumor immune evasion.

## Author Contributions

Jimin Li and Congcong Wu: cohort design, manuscript writing, and data analysis. Yongming Song and Yuhui Fan: statistical analysis and figure preparation. Chao Li: Compilation of clinical information. Shuangping Zhang and Haibo Li: research supervision, manuscript review, and funding support.

## Conflicts of Interest

The authors declare no conflicts of interest.

## Data Availability

The data are available from the corresponding author on reasonable request.

## References

[1] J. Li , J. Xu , Y. Zheng , et al., “Esophageal Cancer: Epidemiology, Risk Factors and Screening,” Chinese Journal of Cancer Research 33, no. 5 (2021): 535. 34815628 10.21147/j.issn.1000-9604.2021.05.01 PMC8580797

[2] R. J. Kelly , “Emerging Multimodality Approaches to Treat Localized Esophageal Cancer,” Journal of the National Comprehensive Cancer Network 17, no. 8 (2019): 1009–1014. 31390584 10.6004/jnccn.2019.7337

[3] A. Rizzo , V. Mollica , A. Marchetti , et al., “Adjuvant PD‐1 and PD‐L1 Inhibitors and Relapse‐Free Survival in Cancer Patients: The MOUSEION‐04 Study,” Cancers 14, no. 17 (2022): 4142. 36077679 10.3390/cancers14174142 PMC9455029

[4] R. J. Kelly , J. A. Ajani , J. Kuzdzal , et al., “Adjuvant Nivolumab in Resected Esophageal or Gastroesophageal Junction Cancer,” New England Journal of Medicine 384, no. 13 (2021): 1191–1203. 33789008 10.1056/NEJMoa2032125

[5] T. K. Sahin , A. Rizzo , S. Aksoy , and D. C. Guven , “Prognostic Significance of the Royal Marsden Hospital (RMH) Score in Patients With Cancer: A Systematic Review and Meta‐Analysis,” Cancers 16, no. 10 (2024): 1835. 38791914 10.3390/cancers16101835 PMC11120545

[6] T. K. Sahin , R. Ayasun , A. Rizzo , and D. C. Guven , “Prognostic Value of Neutrophil‐to‐Eosinophil Ratio (NER) in Cancer: A Systematic Review and Meta‐Analysis,” Cancers 16, no. 21 (2024): 3689. 39518127 10.3390/cancers16213689 PMC11545344

[7] J.‐Y. Hwang , H.‐S. Chen , P.‐K. Hsu , et al., “A Propensity‐Matched Analysis Comparing Survival After Esophagectomy Followed by Adjuvant Chemoradiation to Surgery Alone for Esophageal Squamous Cell Carcinoma,” Annals of Surgery 264, no. 1 (2016): 100–106. 26649580 10.1097/SLA.0000000000001410

[8] L. Zhu , R. Xu , L. Yang , et al., “Minimal Residual Disease (MRD) Detection in Solid Tumors Using Circulating Tumor DNA: A Systematic Review,” Frontiers in Genetics 14 (2023): 1172108. 37636270 10.3389/fgene.2023.1172108 PMC10448395

[9] K. M. Mahuron and Y. Fong , “Applications of Liquid Biopsy for Surgical Patients With Cancer: A Review,” JAMA Surgery 159, no. 1 (2024): 96–103. 37910091 10.1001/jamasurg.2023.5394

[10] J.‐T. Zhang , S.‐Y. Liu , W. Gao , et al., “Longitudinal Undetectable Molecular Residual Disease Defines Potentially Cured Population in Localized Non‐Small Cell Lung Cancer,” Cancer Discovery 12, no. 7 (2022): 1690–1701. 35543554 10.1158/2159-8290.CD-21-1486 PMC9394392

[11] D. Kotani , E. Oki , Y. Nakamura , et al., “Molecular Residual Disease and Efficacy of Adjuvant Chemotherapy in Patients With Colorectal Cancer,” Nature Medicine 29, no. 1 (2023): 127–134. 10.1038/s41591-022-02115-4 PMC9873552 36646802

[12] I. Fernández‐Miranda , L. Pedrosa , M. Llanos , et al., “Monitoring of Circulating Tumor DNA Predicts Response to Treatment and Early Progression in Follicular Lymphoma: Results of a Prospective Pilot Study,” Clinical Cancer Research 29, no. 1 (2023): 209–220. 36269794 10.1158/1078-0432.CCR-22-1654 PMC9811164

[13] R. L. Siegel , K. D. Miller , H. E. Fuchs , and A. Jemal , “Cancer Statistics, 2022,” CA: A Cancer Journal for Clinicians 72, no. 1 (2022): 7–33. 35020204 10.3322/caac.21708

[14] R. L. Siegel , K. D. Miller , and A. Jemal , “Cancer Statistics, 2019,” CA: A Cancer Journal for Clinicians 69, no. 1 (2019): 7–34. 30620402 10.3322/caac.21551

[15] H. Sung , J. Ferlay , R. L. Siegel , et al., “Global Cancer Statistics 2020: GLOBOCAN Estimates of Incidence and Mortality Worldwide for 36 Cancers in 185 Countries,” CA: A Cancer Journal for Clinicians 71, no. 3 (2021): 209–249. 33538338 10.3322/caac.21660

[16] T. D. Azad , A. A. Chaudhuri , P. Fang , et al., “Circulating Tumor DNA Analysis for Detection of Minimal Residual Disease After Chemoradiotherapy for Localized Esophageal Cancer,” Gastroenterology 158, no. 3 (2020): 494–505.e6. 31711920 10.1053/j.gastro.2019.10.039 PMC7010551

[17] D. W. Cescon , S. V. Bratman , S. M. Chan , et al., “Circulating Tumor DNA and Liquid Biopsy in Oncology,” Nature Cancer 1, no. 3 (2020): 276–290. 35122035 10.1038/s43018-020-0043-5

[18] J. Yang , Y. Gong , V. K. Lam , et al., “Deep Sequencing of Circulating Tumor DNA Detects Molecular Residual Disease and Predicts Recurrence in Gastric Cancer,” Cell Death & Disease 11, no. 5 (2020): 346. 32393783 10.1038/s41419-020-2531-z PMC7214415

[19] M. Lipsyc‐Sharf , E. C. De Bruin , K. Santos , et al., “Circulating Tumor DNA and Late Recurrence in High‐Risk Hormone Receptor‐Positive, Human Epidermal Growth Factor Receptor 2‐Negative Breast Cancer,” Journal of Clinical Oncology 40, no. 22 (2022): 2408–2419. 35658506 10.1200/JCO.22.00908 PMC9467679

[20] G. J. Riely , D. E. Wood , D. S. Ettinger , et al., “Non‐Small Cell Lung Cancer, Version 4.2024, NCCN Clinical Practice Guidelines in Oncology,” Journal of the National Comprehensive Cancer Network 22, no. 4 (2024): 249–274. 38754467 10.6004/jnccn.2204.0023

[21] H. Y. Ng , J. M. Y. Ko , K. O. Lam , et al., “Circulating Tumor DNA Dynamics as Prognostic Markers in Locally Advanced and Metastatic Esophageal Squamous Cell Carcinoma,” JAMA Surgery 158, no. 11 (2023): 1141–1150. 37728901 10.1001/jamasurg.2023.4395 PMC10512170

[22] T. Liu , Q. Yao , and H. Jin , “Plasma Circulating Tumor DNA Sequencing Predicts Minimal Residual Disease in Resectable Esophageal Squamous Cell Carcinoma,” Frontiers in Oncology 11 (2021): 616209. 34094900 10.3389/fonc.2021.616209 PMC8173109

[23] T. V. Henriksen , T. Reinert , E. Christensen , et al., “The Effect of Surgical Trauma on Circulating Free DNA Levels in Cancer Patients—Implications for Studies of Circulating Tumor DNA,” Molecular Oncology 14, no. 8 (2020): 1670–1679. 32471011 10.1002/1878-0261.12729 PMC7400779

[24] E. Ococks , A. M. Frankell , N. Masque Soler , et al., “Longitudinal Tracking of 97 Esophageal Adenocarcinomas Using Liquid Biopsy Sampling,” Annals of Oncology 32, no. 4 (2021): 522–532. 33359547 10.1016/j.annonc.2020.12.010

[25] N. Verschoor , M. K. Bos , E. Oomen‐de Hoop , et al., “A Review of Trials Investigating ctDNA‐Guided Adjuvant Treatment of Solid Tumors: The Importance of Trial Design,” European Journal of Cancer 207 (2024): 114159. 38878446 10.1016/j.ejca.2024.114159

[26] T. Reinert , T. V. Henriksen , E. Christensen , et al., “Analysis of Plasma Cell‐Free DNA by Ultradeep Sequencing in Patients With Stages I to III Colorectal Cancer,” JAMA Oncology 5, no. 8 (2019): 1124–1131. 31070691 10.1001/jamaoncol.2019.0528 PMC6512280

[27] P. J. Vellanki , S. Ghosh , A. Pathak , et al., “Regulatory Implications of ctDNA in Immuno‐Oncology for Solid Tumors,” Journal for Immunotherapy of Cancer 11, no. 2 (2023): e005344. 36796877 10.1136/jitc-2022-005344 PMC9936292

[28] S. P. Kerkar and N. P. Restifo , “Cellular Constituents of Immune Escape Within the Tumor Microenvironment,” Cancer Research 72, no. 13 (2012): 3125–3130. 22721837 10.1158/0008-5472.CAN-11-4094 PMC6327310

[29] S. K. Kim and S. W. Cho , “The Evasion Mechanisms of Cancer Immunity and Drug Intervention in the Tumor Microenvironment,” Frontiers in Pharmacology 13 (2022): 868695. 35685630 10.3389/fphar.2022.868695 PMC9171538

[30] D. Ribatti , R. Tamma , and T. Annese , “Epithelial‐Mesenchymal Transition in Cancer: A Historical Overview,” Translational Oncology 13, no. 6 (2020): 100773. 32334405 10.1016/j.tranon.2020.100773 PMC7182759

